# Chemical Modification of Poly(butylene *trans*-1,4-cyclohexanedicarboxylate) by Camphor: A New Example of Bio-Based Polyesters for Sustainable Food Packaging

**DOI:** 10.3390/polym13162707

**Published:** 2021-08-13

**Authors:** Giulia Guidotti, Gianfranco Burzotta, Michelina Soccio, Massimo Gazzano, Valentina Siracusa, Andrea Munari, Nadia Lotti

**Affiliations:** 1 Civil, Chemical, Environmental and Materials Engineering Department, University of Bologna, Via Terracini 28, 40131 Bologna, Italy; giulia.guidotti9@unibo.it (G.G.); gianfranco.burzotta@studio.unibo.it (G.B.); andrea.munari@unibo.it (A.M.); nadia.lotti@unibo.it (N.L.); 2 Interdepartmental Center for Industrial Research on Advanced Applications in Mechanical Engineering and Materials Technology, CIRI-MAM, University of Bologna, 40126 Bologna, Italy; 3 Institute of Organic Synthesis and Photoreactivity, ISOF-CNR, Via Gobetti 101, 40129 Bologna, Italy; massimo.gazzano@unibo.it; 4 Department of Chemical Science (DSC), University of Catania, Viale A. Doria 6, 95125 Catania, Italy; 5 Interdepartmental Center for Agro-Food Research, CIRI-AGRO, University of Bologna, 40126 Bologna, Italy

**Keywords:** *trans*-1,4-cyclohexanedicarboxylic acid, (1R,3S)-(+)-camphoric acid, random copolymers, thermal properties, microstructure, mechanical properties, gas barrier properties, Baur’s equation

## Abstract

Among the several actions contributing to the development of a sustainable society, there is the eco-design of new plastic materials with zero environmental impact but that are possibly characterized by properties comparable to those of the traditional fossil-based plastics. This action is particularly urgent for food packaging sector, which involves large volumes of plastic products that quickly become waste. This work aims to contribute to the achievement of this important goal, proposing new bio-based cycloaliphatic polymers based on *trans*-1,4-cyclohexanedicarboxylic acid and containing different amount of camphoric acid (from 0 to 15 mol %), a cheap and bio-based building block. Such chemical modification was conducted in the melt by avoiding the use of solvents. The so-obtained polymers were processed in the form of films by compression molding. Afterwards, the new and successfully synthesized random copolymers were characterized by molecular (NMR spectroscopy and GPC analysis), thermal (DSC and TGA analyses), diffractometric (wide angle X-ray scattering), mechanical (through tensile tests), and O_2_ and CO_2_ barrier point of view together with the parent homopolymer. The article aims to relate the results obtained with the amount of camphoric moiety introduced and to present, the different microstructure in the copolymers in more detail; indeed, in these samples, a different crystalline form developed (the so-called β-PBCE). This latter form was the kinetically favored and less packed one, as proven by the lower equilibrium melting temperature determined for the first time by Baur’s equation.

## 1. Introduction

In recent years, the growing attention to the increasingly complex and serious problem of waste management and the push by governments to develop the so-called circular economy has required the growingly careful choice of the various plastic materials that are used for various daily purposes. In particular, the packaging sector, both rigid and flexible, absorbs about 40% of the world’s production of plastics, which is around 368 million tons per year [1,2]. Considering the recent development of the economies of many countries worldwide, it is expected that these numbers will grow further and inexorably in the coming decades, and, in the absence of strategic plans for the management of waste production and disposal, will aggravate the already severe problem of environmental pollution. In addition, the principles of circular economy and green chemistry require a low environmental impact of the entire life cycle of materials, starting from raw materials and production processes up to disposal.

Therefore, in recent years, a huge amount of research has been directed towards the individuation for alternative sources of monomers for the synthesis of new plastics. The choice fell onto renewable sources as an alternative to fossil ones, which can give life to eco-sustainable materials, the so-called bioplastics, obtained through green synthetic processes limiting the use of solvents and low greenhouse gas emissions.

Furthermore, the development of flexible packaging in place of rigid one, especially in the food sector, and can lead to a significant decrease in plastic product volumes, and therefore, in the material that in the short term will become waste. 

The versatility and suitability of monomers, from renewable resources or even from agri-food waste, make aliphatic polyesters one of the most important classes of synthetic polymers and are currently being proposed for packaging, automotive, and pharmaceutics, just to cite some of the fields in which these materials are intensively used. However, they rarely meet satisfactory thermomechanical properties for applications such as packaging, films, or fibers [3].

Cycloaliphatic-based polyesters are particularly interesting, as they are characterized by intermediate physical properties with respect to aromatic and linear aliphatic polyesters, with the exception of yellowing resistance, which is similar to that of polymers from linear diacids [4]. In detail, their glass transition temperature is lower than that of aromatic polyesters but higher than the one of linear aliphatic polyesters. Their flexibility is also intermediate due to their cyclic structure. In case of 1,4-cyclohexanedicarboxylic acid (1,4-CHDA)-based polyesters, such a trend can be explained on the basis of the energy absorption due to the interconversion of the chair and boat conformations. Compared to terephthalic acid (TPA)-based polyesters, those obtained from 1,4-CHDA show improved processability, having a reduced melt viscosity; higher photo-oxidative stability, as the phenyl ring readily absorbs UV-light; and higher thermal stability [4,5,6]. Moreover, 1,4-CHDA-based polyesters provided better humidity and impact resistance, keeping the compostability characteristic of linear aliphatic polyesters preserved [7,8]. Heidt and Elliot [9] investigated the effects of introduction in the isophthalic-based polyesters of adipic acid (AA) or 1,4-CHDA. Both 1,4-CHDA and AA flexibilised the polymer chain to the same extent, but in the case of cycloaliphatic diacid, the hardness and T_g_ value were preserved.

1,4-cyclohexanedicarboxylic acid is currently mainly produced as an oil-based derivate, but a green route is also available [10]. 

Considering the good resistance to heat, light, and humidity, poly(alkylene 1,4-cyclohexanedicarboxylate)s are promising candidates for packaging applications. Of particular interest is poly(butylene *trans*-1,4-cyclohexanedicarboxylate) (PBCE), characterized by smart gas barrier performances that are comparable to those of PET and PLA and superior to those of polyolefins, even though they are too rigid for the production of transparent and flexible films due to their high crystallization rate. Such drawbacks have been overcome by copolymerization with suitable counits [7,11,12,13,14].

Camphoric acid is another bio-based monomer consisting of a conformationally rigid five-membered aliphatic ring that is obtainable from the oxidation of bicyclic terpene (1R)-(+)-camphor by nitric acid. Camphor, in turn, can be obtained from wood distillation of camphor tree (*Cinnamomum camphora*), originally widespread in Borneo, Taiwan, and eastern Africa. Nowadays, it is also cultivated in many other parts of the world, including North America [15,16]. This cheap monomer is already widely present on the market, exceeding USD 100 million a year [17,18]. Given the high demand, about 17,000 tons are produced each year through the precursor α-pinene [19,20,21,22], which can be easily extracted from wood pulp, at a cost of about USD 3/kg [23]. Compounds deriving from camphor are mainly used as chiral precursors in enantioselective organic syntheses [24,25,26,27].

Camphor can also be considered as an interesting building block for the production of novel bio-based polymers, as documented by several studies on camphor-based polymers that have recently been reported in the literature [28,29,30,31,32,33,34].

In particular, in 2019, Nsengiyumva and Miller [35] synthesized and characterized a series of high molecular weight polyesters (about 20,000 Da) containing camphoric acid and different diols, both linear and cyclic from the molecular and thermal point of view. Poly(ethylene camphorate) (PEC) showed characteristics comparable to those of PLA. The polymers containing cyclic diols showed T_g_ values higher than PEC, while those synthesized starting from diols containing longer aliphatic chains presented lower T_g_ values. Preliminary biodegradability studies in acidic water solution conducted on PEC indicated a good biodegradability. Furthermore, PEC-PET copolymers, prepared starting from bis-hydroxyethyl camphorate (BHEC) and bis-hydroxyethyl terephthalate (BHET), were investigated. Camphorate introduction enhanced the bio-based content of the resultant polymer but also decreased the T_g_ value with respect to PET [35].

Another interesting building block is represented by (1R,3S)-1,2,2-trimethylcyclopentane-1,3-dimethanol (TCDM), a rigid alicyclic diol prepared by the oxidation and the subsequent reduction of natural camphor. TCDM has been employed together with linear α,ω-diacids or terephthalic acid (TPA) or 2,5-furandicarboxylic acid (FDCA) for the synthesis of a series of novel bio-based polyesters with interesting functional properties. The polyester based on TCDM and TPA was characterized by high thermostability, a high T_g_ value (over 100 °C), a high elastic modulus, and stress at break; the polyester based on TCDM and 1,4-succinic acid exhibited excellent ductility and resilience [36]. 

Finally, a series of PBS-based copolymers characterized by both random and block architecture were synthesized starting from a 1,4-butanediol/TCDM mixture and succinic acid by bulk polycondensation and reactive mixing, respectively. The introduction of rigid TCDM moiety along the PBS polymer chain improved PBS thermal stability, increased glass transition temperature, and improved mechanical properties, the degree of improvement being more significant for block copolymers. The camphor-based diol also enhanced the hydrolytic degradation of PBS [37]. 

To the best of our knowledge, nobody has chemically modified PBCE by introducing camphor moiety along its macromolecular chain so far. 

As mentioned before, poly(butylene *trans*-1,4-cyclohexanedicarboxylate) is highly chemically and thermally stable. Nevertheless, it is quite opaque and fragile due to the degree of crystallinity. Copolymerization with camphoric acid could improve the unsatisfying characteristics without compromising the already good ones. Besides being cheap and bio-based, camphoric acid contains a cycloaliphatic moiety; thus, it is supposed to preserve the chemical and thermal stability of PBCE. Moreover, the introduction of camphoric acid could reduce the PBCE crystallinity degree and thus the fragility and opacity of the final material.

Such chemical modification was conducted in the melt, avoiding the use of solvents. The so-obtained polymers have been processed in the form of films by compression molding. The thermal, structural, mechanical, and gas barrier properties have been investigated and correlated to the amount of camphoric moiety that was introduced. 

## 2. Materials and Methods

### 2.1. Materials

(1R,3S)-(+)-Camphoric acid (CA) (J&K Scientific), trans-1,4-cyclohexanedicarboxylic acid (1,4-CHDA) (Fluorochem), 1,4-butanediol (BD), titanium tetrabutoxide (TBT) and titanium isopropoxide (TIP) (Sigma Aldrich) were all used as purchased.

### 2.2. Synthesis

**Scheme 1:**
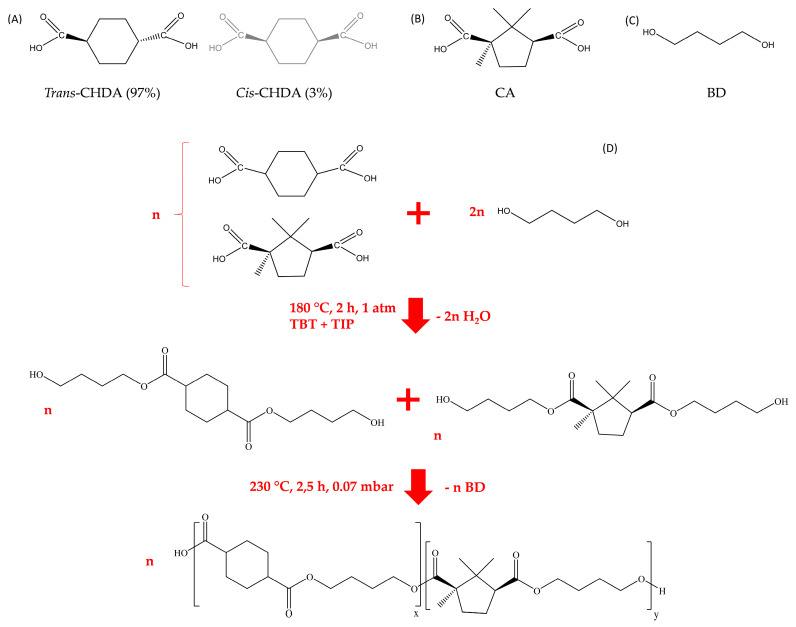
Chemical structure of (**A**) trans- and cis-1,4-cyclohexanedicarboxylic acid; (**B**) (1R,3S)-(+)-camphoric acid and (**C**) butanediol; (**D**) schematic representation of polymer synthesis.

The syntheses of poly(butylene trans-1,4-cyclohexanedicarboxylate) homopolymer (PBCE) and poly(butylene trans-1,4-cyclohexanedicarboxylate/camphorate) copopolymers (P(BCE_x_BC_y_)), with x and y indicating the molar percentage of butylene trans-1,4-cyclohexanedicarboxylate and butylene camphorate counits, respectively), described in Scheme 1D, were conducted through two-stage melt polycondensation reactions starting from 1,4-CHDA in the case of PBCE and a 1,4-CHDA/CA mixture in the case of the copolymers, and BD, which was later used in a molar excess of 100%, in order to favour both the solubilization, mixing, and esterification reactions of the two diacids. In more detail, during the first phase (T = 180 °C, inert nitrogen atmosphere, 100 rpm), all of the reagents were inserted into a glass reactor together with the catalysts. A mixture of TBT and TIP (200 ppm/g of final polymer of each, about 12.8 mg and 11 mg, respectively) was used as a catalyst. During this phase, which lasted a total of two hours, i.e., until 90% of the theoretical amount of water was distilled off, direct esterification reactions occurred with the elimination of the water molecules. In more detail, each diacid molecule reacted with two butanediol ones (n moles of diacid mixture react with 2n moles of glycol), leading to the formation of BDO-diacid-BDO moieties. Afterwards, during the second stage, the temperature was set to 200 °C, and a gradually increasing vacuum up to 0.07 mbar was applied in order to facilitate the transesterification reactions between BDO–diacid–BDO moieties and the removal of the glycolic excess, which occurred through distillation. During this phase, the torque value became progressively higher, indicating the increase in the length of molecules, starting from dimers, trimers, oligomers up to high molecular weight. At the end of this second step, which lasted until a constant torque value was reached (about 2.5 h), the system was brought to a temperature of 230 °C for 5 additional minutes. The obtained polymers were then discharged from the reactor and were purified by solubilization in chloroform and subsequent precipitation in cold methanol.

### 2.3. Molecular Characterization

^1^H-NMR analysis was performed to check the chemical structure and the chemical composition of the synthesized polymers. The ^1^H-NMR spectra were acquired using a Varian XL-400 NMR spectrometer (Palo Alto, CA, USA) at room temperature (relaxation time = 0 s, acquisition time = 1 s, 100 repetitions). The polymeric solutions were prepared by dissolving 10 mg of material in deuterated chloroform (containing 0.03% TMS as internal reference) with a concentration of 0.5 wt%.

To determine molecular weights, GPC analysis was conducted at 30 °C with an HPLC 1100 chromatograph (Agilent Technologies, Santa Clara, CA, USA) equipped with a Plgel 5-mm MiniMIX-C column. The chromatograms were recorded with HP Chemstation version A.05.04 and were processed with the GPC Calculator 7.6 (General Electric Company) software. The eluent used was chloroform (column flow 0.3 mL/min), and the injected solutions had a concentration of 2 mg/mL. The molecular weights were calculated using a 3rd order calibration curve obtained from polystyrene standards with a molecular weight ranging between 2000 and 100,000 Da.

### 2.4. Films by Compression Moulding

Before proceeding with further characterization, compression moulded polymeric films (about 100 μm thick) were obtained by means of a Carver laboratory press (Wabash, IN, USA) equipped with aluminum heating plates. A total of 2.5 g of each sample was heated in between two Teflon sheets at a temperature 30 °C higher than the melting temperature and were held for 2 min before applying a pressure of 5 ton/m^2^ for 1 min. The films were then ballistically cooled down in a press. The obtained films were stored at room temperature for 3 weeks in order to let them reach equilibrium crystallinity. 

### 2.5. Thermal and Structural Characterization

Thermogravimetric analysis (TGA) was performed to determine the temperature of initial degradation (T_onset_) and the temperature corresponding to the maximum weight loss rate (T_max_). The measurements were conducted using a Perkin Elmer (Shelton, CT, USA) TGA4000 instrument under inert nitrogen atmosphere (40 mL/min) and heating weighed samples of about 5 mg at a constant rate of 10 °C/min in the temperature range of 40–800 °C.

DSC analysis was performed to determine the main thermal transition in the polymer when subjected to a predetermined heating program. Measurements were conducted with a Pyris DSC6 calorimeter (Perkin Elmer, Shelton, CT, USA) under nitrogen flow (20 mL/min) using the following thermal program: (a) the polymer was brought to a temperature of −70 °C and was subsequently heated at a rate of 20 °C/min until it reached a temperature 40 °C higher than its melting temperature (T_m_) (I scan); (b) the polymer was kept at this temperature for 3 min and then was rapidly cooled down to −70 °C at a rate of 100 °C/min in order to limit any crystallization phenomena as much as possible; (c) a second scan similar to the first one was performed at the same heating rate (II scan). The glass transition temperature (T_g_) was calculated as the midpoint of the glass-to-rubber transition step, while the specific heat increment (ΔC_p_) was obtained from the jump height between the two baselines associated with the glass-transition step. T_m_ and the corresponding heat of fusion (ΔH_m_) were determined as the peak maximum of the endothermic phenomenon in the DSC curve and the total area of this endothermic signal, respectively. 

In order to determine the kind and amount of crystalline phases developed by the materials under investigation, Wide Angle X-ray Scattering (WAXS) analysis was performed by means of a X’Pert PANalytical diffractometer (Almelo, The Netherlands) using the wavelength of the Kα radiation of copper (λ = 15418 Å) equipped with a solid state detector X’Celerator (Almelo, The Netherlands). The 2θ interval from 5° to 60° was explored (step of 0.1° and counting time of 100 s/point). The crystallinity degree (X_c_) was calculated from the diffraction profile by dividing the area related to the fraction of crystalline material (A_c_) by the total area underlying the diffraction profile (A_t_) according to the following equation:

X_c_ = A_c_/A_t_ (1)

A_c_ was obtained by subtracting the portion due to the amorphous material from the whole diffraction profile, which was modeled as a bell-shaped peak.

### 2.6. Mechanical Characterization

All of the polymers six synthesized specimens (50 mm × 5 mm) for each sample were characterized from a mechanical point of view through tensile stress–strain tests in order to evaluate elastic modulus (E), elongation at break (ε_B_), and stress at break (σ_B_). After having measured the average thickness, these specimens were fixed to the instrument, an Instron 5966 dynamometer (Norwood, MA, USA), with a transducer-coupled 10 kN loading cell controlled by a computer. A 20 mm gauge length used was. The tests were conducted at room temperature with a strain rate of 10 mm/min. The obtained results are reported as mean value ± standard deviation. For the determination of E, the initial linear slope of the stress–strain curves was considered.

### 2.7. Gass Barrier Properties Evaluation

Since gas permeability is a key parameter that must be evaluated in the case of applications in the packaging field and in the food packaging field in particular, the determination of the gas transmission rate (GTR) of the O_2_ and CO_2_ through the polymeric films (diameter of 10 cm) was determined. For this purpose, a GDP-C Permeabilimeter (Brugger Feinmechanik GmbH, München, Germany) was used with a manometric method, according to the ASTM 1434-82 standard (Standard test Method for Determining Gas Permeability Characteristics of Plastic Film and Sheeting), DIN 53 536 in compliance with ISO/DIS 15 105-1, and according to the Gas Permeability Testing Manual (Registergericht München HRB 77020, Brugger Feinmechanik GmbH). The tests were performed at 23 °C using food-grade gases (flow rate of 100 cm^3^/min) in dry conditions (RH = 0%) on a sample area of 78.5 cm^2^ (standard measurement area). The sample temperature was set by an external thermostat HAAKE-Circulator DC10-K15 type (Thermo Fisher Scientific, Waltham, MA, USA). Permeability measurements were performed at least in triplicate, and the mean value is presented. 

Film thickness was calculated with a digital micrometer (MarCator type 1086, Mahr GmbH, Esslingen, Germany) connected to a PC containing the software *sample thickness tester DM-G*. The reported results represent the mean value thickness of three experimental tests run at 10 different points on the polymer film surface at room temperature.

## 3. Results and Discussion

### 3.1. Molecular Characterization

The copolymer objects of the study were synthesized starting from two different diacids: 1,4-cyclohexanedicarboxylic acid (1,4-CHDA) (Scheme 1A) and (1R,3S)-(+)camphoric acid (CA) (Scheme 1B) together with 1,4-butanediol (BD) (Scheme 1C). A schematic representation of the synthetic procedure is reported in Scheme 1D.

**Scheme 2:**
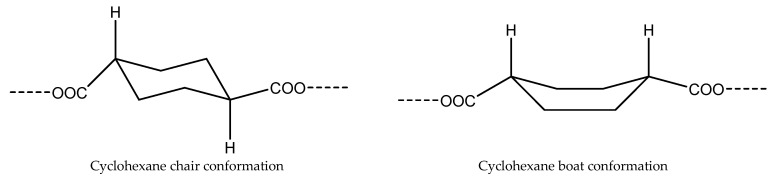
Chair and boat conformations of cyclohexane moiety.

As one can see, 1,4-cyclohexanedicarboxylic acid contains a six-membered aliphatic cycle with two carboxylic groups in the 1,4-position and is mainly composed of trans-1,4-cyclohexanedicarboxylic acid isomer (97%) and of a negligible amount of cis-1,4-cyclohexanedicarboxylic acid isomer (3%). Isomerism deeply affects the most stable cyclohexane moiety conformation. In more detail, in the case of trans isomers, cyclohexane preferentially adopts the chair conformation, while in cis isomers, the boat formation is adopted (Scheme 2). Concerning camphoric acid, it contains a five-membered aliphatic cycle with two carboxylic groups in 1,3-position, two methyl groups on C_1_, and one -CH_3_ on C_3_. Camphoric acid presents a defined configuration at the two stereocenters (1R,3S).

The PBCE homopolymer together with its camphoric-containing P(BCE_x_CE_y_) copolymers were subjected to molecular characterization that allowed the chemical structure, molar composition (^1^H-NMR) and molecular weight (GPC) to be checked.

**Figure 1:**
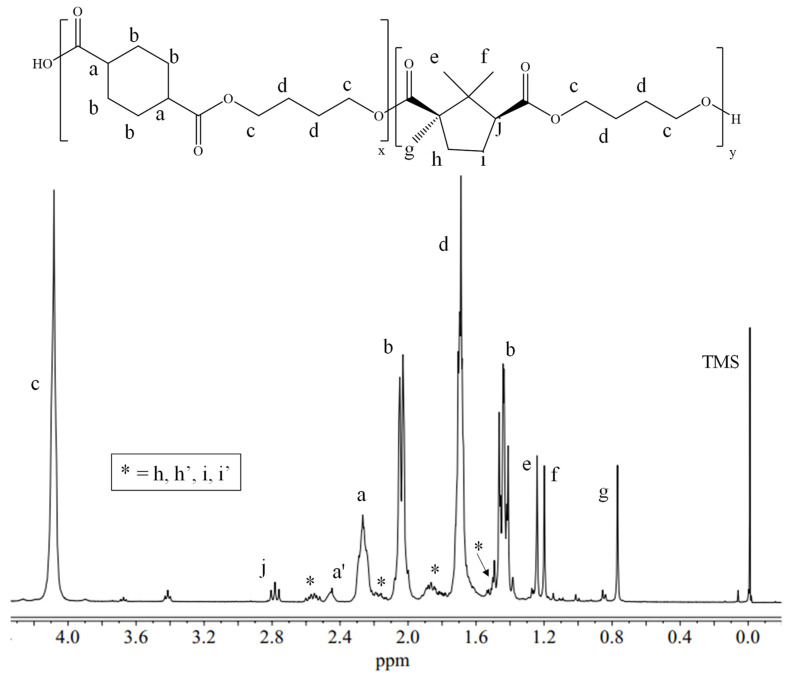
^1^H-NMR spectrum of P(BCE_85_BCE_15_) copolymer with the corresponding peak assignment.

The obtained spectra confirm the expected structure. As an example, in Figure 1, the ^1^H-NMR spectrum of P(BCE_85_CE_15_) together with the chemical structure and peak assignment is shown.

In addition to the signals of d-chloroform and tetramethylsilane used as reference, one can detect the peaks related to cyclohexane moiety: a (2.33 ppm), a’ (2.45 ppm), and b (2.05 and 1.42 ppm); signals c and d show peaks at 4.11 and 1.68 ppm of the butylene subunit; the peaks of camphoric ring and in particular, those of the e, f, and g signals relating to the protons of the three methyl groups at 1.22, 1.20 and 0.78 ppm, respectively; the j peak at 2.79 ppm coming from the protons on the carbon atom adjacent to the carboxylic group; and finally, the multiple signals h, h’, I, and i’ of the methylene protons of the ring placed at the same distance from each other at 2.55, 2.18, 1.85, and 1.49 ppm, respectively.

The actual molar composition was determined from the normalized area underlying the j peak of the camphoric subunit and the area of a and a’ signals of the cyclohexane moiety. It should be highlighted that the contribution of the * signal due to the camphoric subunit and that was located at fields immediately higher (2.20 ppm) was subtracted from the area of a peak.

Finally, the percentage of cis-isomer of the 1,4-cyclohexandicarboxylate subunit was calculated from the ratio between the a’ and a signals areas. It was found to be comparable in all cases with values between 4 and 8% and slightly increased with the amount of counit introduced in the PBCE backbone.

As it can be seen from the data reported in Table 1, all of the samples are characterized by quite high molecular weights (M_n_) and a low polydispersity index (D), which was determined through GPC analysis, indicating a good control of the polymerization process for the homopolymer as well as for the copolymers. Although all of the materials reached quite high molecular weight values, the copolymers characterized by higher amounts of camphoric subunit have slightly lower weights. This effect is probably due to the presence of a methyl group on the carbon in α position to the carboxylic group of camphoric moiety, which makes the electrophilic carboxylic carbon less accessible to nucleophilic attack of the hydroxyl group of the glycol unit because of its higher steric hindrance than the H atom.

### 3.2. Thermal and Structural Characterization

**Figure 2:**
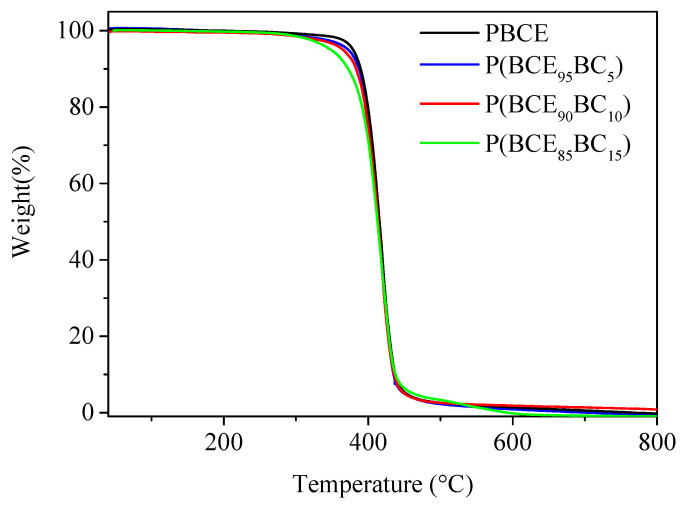
TGA traces of PBCE and P(BCE_x_BC_y_) copolymers. Heating rate: 10 °C/min under N_2_ flow.

The thermal stability of all of the samples was studied by the TGA analysis conducted under nitrogen flow. From the thermogravimetric curves (shown in Figure 2), it was possible to determine the temperature corresponding to the weight loss onset (T_onset_) and that of the maximum degradation rate (T_max_), reported in Table 2. All of the polymers that were studied showed excellent thermal stability, weight loss starting from temperatures between 385 and 395 °C, that regularly decreased with the content of the camphoric subunit. An explanation of this trend can be found in the higher ring strain of a five-membered ring (camphoric subunit) with respect to cyclohexane, which in turn makes the camphoric moiety more reactive and consequently less thermally stable. Furthermore, considering that the ring strain values are 6.2 and 0.1 Kcal/mol [38], it is reasonable that the cyclopentane starts degrading at lower temperature than the cyclohexane, as it is more constrained and tensioned and therefore subject to easier breakage under heating. Summarizing, we can consider the different ring strain of the 6C-membered cycle (cyclohexane) and of the 5C-membered one (camphor) as main reason for the slight thermal stability decrease. As already mentioned, while the T_onset_ value slightly decreases, T_max_ remains practically constant above 400 °C, indicating that in all cases, the main degradation mechanism is the same and is characterized by the breakage of the carboxylic groups and the PE-like segments present in both counits. One can assert that copolymerization did not particularly affect the excellent thermal stability of the PBCE homopolymer, which is one of its strengths. Finally, as it can be seen from Figure 2, in all cases, the weight loss is practically 100%.

**Figure 3:**
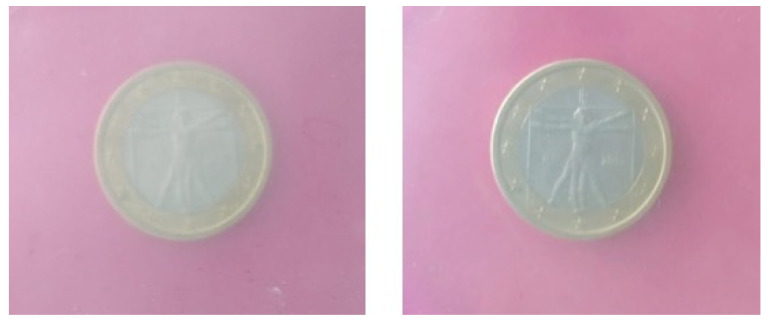
Pictures of PBCE homopolymer (**left**) and P(BCE_85_BC_15_) copolymer (**right**).

The materials under study have also been subjected to differential scanning calorimetry (DSC) and wide angle X-ray scattering (WAXS) analyses. These techniques have been conducted on both the powders obtained through purification process and the free-standing films obtained by compression molding (see, as example, which can be seen in Figure 3 PBCE and P(BCE_85_BC_15_) films), as described in the Experimental Section. Before characterization, the films have been stored at 25 °C for three weeks to reach equilibrium crystallinity.

As it can be noted from Figure 3, the film prepared from the P(BCE_85_BC_15_) copolymer is more transparent than the PBCE one.

**Figure 4:**
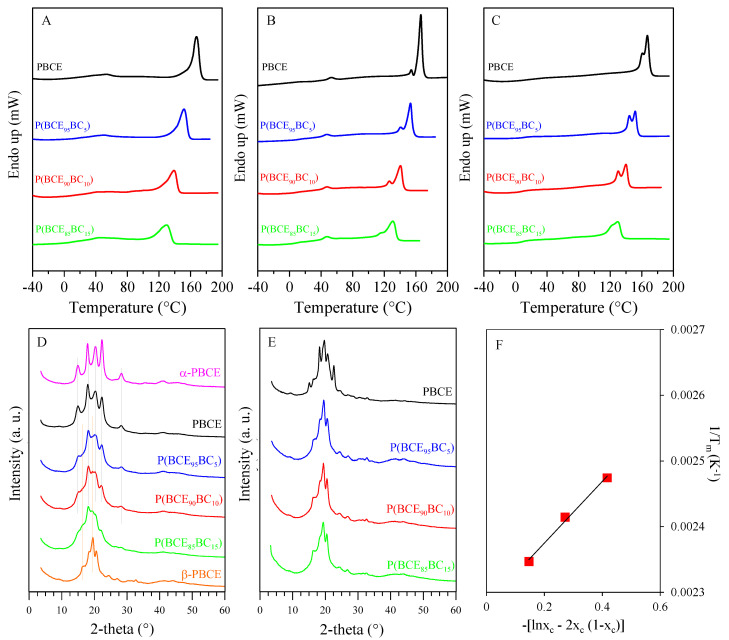
Calorimetric curves (heating rate: 20 °C/min) and diffraction profiles (WAXS) of the PBCE and related P(BCE_x_BC_y_) copolymers. (**A**) I scans of powders; (**B**) I scans of films; (**C**) II scans after rapid cooling from the melt; (**D**) WAXS profiles of powders (for PBCE, the diffractograms corresponding to α-PBCE and β-PBCE crystal lattices, previously obtained by different treatment are also shown for comparison; vertical lines are eye guides); (**E**) WAXS profiles of films; (**F**) 1/T_m_ vs. −[lnx_c_ − 2x_c_ (1 − x_c_)] for the P(BCE_x_BC_y_) copolymers. The line represents the theoretical trend according to the Baur equation.

The I and II scan calorimetric curves of the powders and films are shown in Figure 4. In Table 2, the corresponding thermal data are collected.

As it can be seen from the first calorimetric scan curves of the powders (Figure 4A), all of the polymers are semicrystalline: in fact, the corresponding DSC traces show an endothermic deviation at low temperatures, which is associated with the glass transition phenomenon, followed by an endothermic peak at higher temperatures, which is typical of the fusion of the crystalline portion. In addition, in all of the materials, a weak endothermic signal is detected at an approximately constant temperature, around 50 °C, whose area is difficult to determine given the width and the low intensity of the peak. On the other hand, regarding the endothermic melting peak at a higher temperature, one can appreciate a reduction of both T_m_ and ΔH_m_ as the counit content increases. This trend, which is quite typical in copolymers, suggests a lower perfection degree as well as a reduction of the crystalline portion. Both of these effects are due to the hindering of the crystallization ability of the PBCE homopolymer chains as a consequence of the insertion of camphoric subunits. A concern of the first calorimetric scan of the polymers in form of film (Figure 4B) is that the corresponding traces are qualitatively similar to the ones obtained from the powders. Moreover, by looking more in detail, some differences can be highlighted. First, the tiny endothermic peak at the temperature in between T_g_ and T_m_ at an approximately constant temperature (47–53 °C) istime is now more defined and slightly rises in intensity as the content of the BC counits increases. Previous studies [39,40,41] have shown that this phenomenon could be attributable to the isotropization of a one- or two-dimensional ordered array, referred to as the mesophase. The development of this peculiar structure is possible thanks to the presence of mesogenic groups, such as the cyclohexane ring, together with flexible aliphatic units, consisting of the butylene segment. Second, the endothermic phenomena at higher temperatures consist of multiple peaks. This evidence could be related to the melting/crystallization/melting phenomena or to the presence of several crystalline phases. In this view, the WAXS analyses can shed light on the origin of the multiple endotherms.

As is known, a partially crystalline material generally exhibits a different behavior compared to its fully amorphous analogue. Although conflicting results are reported in the literature, crystallinity is believed to act as physical cross-links, causing an increase in T_g_ values. Therefore, to study the dependence of the glass transition on the composition, it is appropriate to examine the phenomenon in the total absence of crystallinity. With the aim of obtaining amorphous specimens, all of the polymers were subjected to fast cooling from the melt. The DSC curves and the thermal characterization data of the so-treated materials are shown in Figure 4C and Table 2, respectively. As it can be seen, the II scan curves of the PBCE and its copolymers show the typical behavior of semicrystalline materials already highlighted in the first scan, demonstrating the high crystallization rate of these polymers, which, under the adopted experimental conditions, cannot be locked in the amorphous state. Additionally, from the DSC traces after rapid cooling from the melt, it can be seen how copolymerization determines a progressive lowering of the melting temperature and the associated heat as the content of camphoric subunit increases. Furthermore, all the traces once again show multiple melting peaks. Regarding the glass transition step, by observing the trend of the T_g_ values, we can evidence a flexibilizing effect reached by copolymerization, as indicated by the progressive lowering of the glass transition temperature as the quantity of BC counits increases, which is accompanied by the enhancement of the amorphous phase fraction (higher Δc_p_ values). This effect has already been observed by Nsengiyumva et al. [35] in a copolymeric system of poly(ethylene terephthalate) containing a camphoric subunit.

As already mentioned, the nature of the crystalline phase present in the polymers under study, both in form of powders and films, has been studied by WAXS. Figure 4D shows the diffraction patterns of the samples in powder form. The polymers show decreasing crystallinity values as the composition changes, with the PBCE being much more crystalline than the copolymers (Table 2). Previous studies [8,42] had highlighted the polymorphic nature of the PBCE homopolymer, whose crystalline lattice can change through copolymerization, even by introducing just small quantities of counits or by different thermal treatment. α and β crystalline lattices were then determined for PBCE (reported for the sake of comparison in Figure 4D). In particular, the α-PBCE phase is characterized by reflections at 2θ angles equal to 14.9°, 17.9°, 20.3°, 22.3°, 28.2°, and 35.7°, while the β-PBCE lattice presents a diagram with peaks located at the 2θ angles of 9.3°, 16.5°, 18.5°, 19.5°, 20.7°, 24.4°, 27.1°, and 32.7°. Diffractometric analysis allows the highlighting of how copolymerization leads to a net decrease in the intensity as well as a widening of the peaks and therefore a reduction in the degree of crystallinity compared to the homopolymer. Although the low X_c_ values make it difficult to identify the type of crystalline phase present, it is possible to hypothesize that for all of the materials in the form of purified powders, there is the simultaneous presence of both crystalline phases (α and β). Furthermore, it seems that the purification process, mainly solvent evaporation, favors the formation of the α-PBCE phase that is present to a larger extent. Nevertheless, it is possible to notice some reflections that are typical of the β-PBCE phase, which become more and more evident as the quantity of counits increases (Figure 4D). As for the compression molded films, Figure 4E shows the corresponding diffractograms. In this case, the samples show crystallinity values (Table 2) that are much more similar to each other, including PBCE homopolymer, only slightly decreasing as the content of the camphoric subunit rises. Contrary to what has been observed for powders, in copolymeric films, it is easier to recognize that the predominant crystalline lattice is the one indicated as β-PBCE. Only in the PBCE omopolymer film are the peaks belonging to the α-phase also detectable. β-PBCE is a unique phase in the copolymers regardless of the composition, indicating how the inclusion of butylene camphorate counits, even if in very small quantities, causes a sudden change of the microstructure. Furthermore, considering that the peaks do not show changes in position nor variations in the relative distances, co-crystallization can be ruled out, i.e., the counit are excluded from the crystalline lattice of the homopolymer.

Finally, we proceeded to verify the applicability of Baur’s equation proposed in the literature [43] to describe the trend of T_m_ with the composition:

(2) $$\frac{1}{T_{m}}=\frac{1}{T_{m}^{0}}-\left[\frac{R}{\Delta H_{m}^{0}}\right]\left(lnx_{c}-2x_{c}\left(1-x_{c}\right)\right)$$

where *T_m_* is the first scan melting temperature, (K) is the copolymer of the film, *x_c_* is the molar fraction of the crystallizable BCE counits, *T_m_*_0_ is the equilibrium melting temperature and Δ*H_m_*^0^ is the the equilibrium melting enthalpy of the homopolymer (in this case PBCE), and *R* is the gas constant. Figure 4F shows the values of *1*/*T_m_* as a function of −[*lnx_c_* − 2*x_c_* (1 − *x_c_*)]. The line indicates the theoretical trend of *T_m_* described according to the Baur’s equation. As one can see, the experimental data of the copolymers, which are the object of this study, fit the theoretical curve very well, with *T_m_* progressively decreasing as the content of BC counits increases. Thus, the exclusion model described by the Baur equation is confirmed in accordance with the diffractometric data. The crystalline phase present in copolymer films is pure, with the BC counits being totally confined in the amorphous phase. Furthermore, Baur‘s equation allows the melting temperature and the heat capacity of the fully crystalline polymer (*T_m_*^0^ and Δ*H_m_*^0^) of the lattice developed in the compression molded films to be calculated. The value obtained for *T_m_*^0^ is 162 °C, while the value of Δ*H_m_*^0^ is equal to 74 J/g, and both values are lower than those calculated for the α-PBCE lattice (thermodynamically more stable), equal to 184 °C and 78 J/g, respectively [11,44]. In light of this evidence, it is reasonable to assume the crystal phase present in the copolymer films is the β-PBCE one, which is kinetically favored and less compact and packed than α-PBCE and therefore characterized by lower *T_m_*^0^ and Δ*H_m_*^0^ values. The applicability of Baur’s equation also corroborates the random distribution of the counits along the macromolecular backbone.

### 3.3. Mechanical Characterization

**Figure 5:**
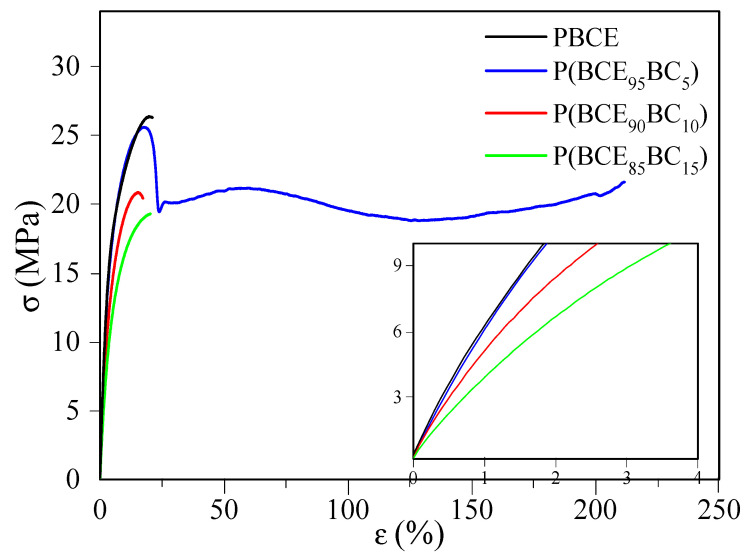
Stress–strain curves of PBCE and P(BCE_x_BC_y_) copolymers.

In order to obtain information on the mechanical properties of the synthesized materials, tensile tests were conducted on PBCE homopolymer and its related copolymers. The obtained stress–strain curves are shown in Figure 5, while the mechanical data (elastic modulus E, stress at break σ_B_, elongation at break ε_B_) are reported in Table 3.

The homopolymer was the most rigid material among those tested, characterized by the highest value of elastic modulus (E) and stress at break (σ_B_). With the introduction of the BC counit, the mechanical response of the materials varies. In fact, a reduction in the elastic modulus and stress at break was detected, and the extent of the variation resulted in the more consistent and greater BC counit content. This result is correlated to the gradual reduction of crystallinity degree and T_g_ in the copolymers with respect to the homopolymer. In particular, the P(BCE_95_BC_5_) copolymer showed a peculiar behavior: it is the only one in which it is possible to detect yielding phenomenon together with quite high elongation at break (ε_B_ ≈ 200%). This significant improvement, achieved through the introduction of a small amount of counit (5 mol%), can be considered as the result of the synergistic contribution of several factors. First of all, the lowering of both the degree of crystallinity and of the T_g_ favors chain flexibility. It should also be remembered that copolymerization, regardless of the amount of counit introduced, determines the development of a crystalline phase (β-PBCE) that is different from the one predominant in the homopolymer (α-PBCE) together with the formation of a mesophase characterized by a different level of packing which, from a mechanical point of view, exerts a flexibilizing effect. Furthermore, previous studies in the literature have shown that the crystalline phase develops at the expense of the mesophase [14,45,46]: it is therefore reasonable that a smaller amount of 3D-orderd microstructure is accompanied by a higher amount of mesophase. This latter aspect, in turn, provides the excellent performance material in terms of elongation at break. As mentioned above, the most significant improvement was observed in the copolymer containing the smallest amount of counits; such results can be explained on the basis of another variable that significantly affects the mechanical properties: molecular weight. Although the molecular weight values are quite high for all the copolymers, P(BCE_95_BC_5_) is the copolymer with the highest molecular weight (Table 1).

As for the elongation at break values of the P(BCE_90_BC_10_) and the P(BCE_85_BC_15_) copolymers, they are comparable to those measured for the homopolymer, with respect to which, however, they are less stiff (lower E). Copolymerization, therefore, allows materials characterized by different mechanical properties suitable for different applications to be obtained.

### 3.4. Gas Barrier Properties

**Figure 6:**
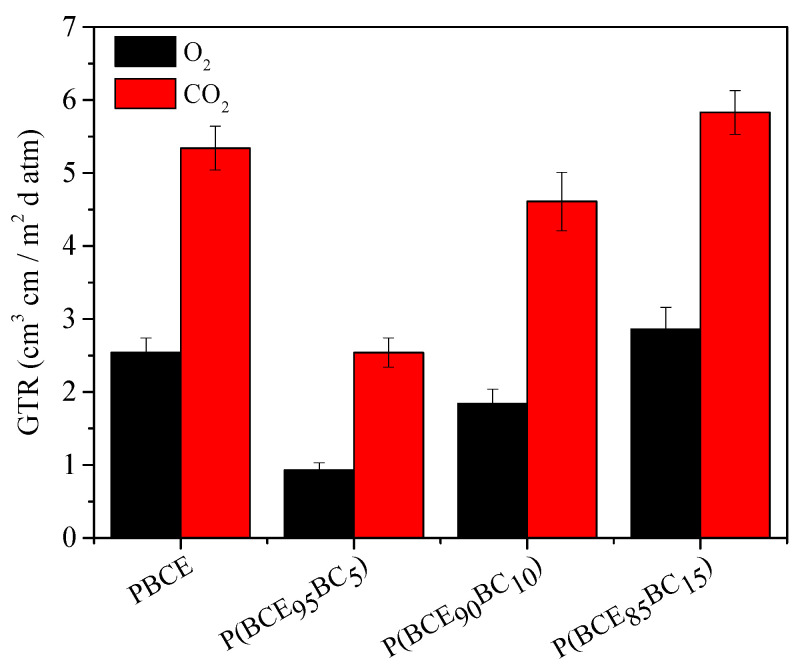
GTR values to dry O_2_ and CO_2_ at 23 °C of PBCE and P(BCExBCy) copolymers.

The barrier properties of the PBCE and its copolymers to dry O_2_ and CO_2_ at 23 °C were evaluated. The permeability values, expressed as the gas transmission rate (GTR) (cm^3^cm/m^2^ d atm), are collected in Table 3 and are presented in Figure 6.

From the analysis of the results, it is evident how the introduction of just small amounts of butylene camphorate counit (5 mol%) in the macromolecular chain of PBCE provides a significant improvement in the gas blocking capability of the copolymer film compared to the homopolymer one; on the contrary, by further increasing the amount of butylene camphorate counit, the enhancement is progressively reduced, leading to GTR valuescomparable of those of the homopolymer for the sample richest in BC moieties. This trend cannot be ascribed to a reduction of amorphous phase mobility since all of the materials under the current operating conditions are in the rubbery state (T_g_ < 23 °C), corresponding to a mobile amorphous phase. In addition, the macromolecular mobility in all of the polymers is comparable and is even slightly higher for the copolymers (T_g_ values reduce). The better performance of P(BCE_95_BC_5_) with respect to PBCE could be related to the presence in the two samples of the different crystalline phases (in the copolymer (β-PBCE lattice), as opposed to in the homopolymer (α- and β-PBCE phases), as highlighted through diffractometric analysis. Furthermore, as already mentioned, the simultaneous presence of rigid subunits (aliphatic cyclic structures) and flexible segments (linear aliphatic chains) allows for the development of a mesophase (presumably originated from the stacking of the cyclohexane rings, which are in the chair conformation), characterized by a very compact packing of the macromolecules, which very efficiently hinders the passage of gases. Mesophase is typically present in liquid–crystalline polymers, known for their exceptional barrier properties [47]. The formation of mesophase generally occurs at the expense of the crystalline phase, so, in the light of the calculated X_c_ values, an increase in the amount of mesophase can be assumed as the counit content increases. As confirmation of that, the calorimetric measurements evidenced by the presence of a low temperature isotropization phenomenon in the copolymers, is possibly ascribable to the mesophase. On the other hand, the inclusion of quantities greater than 5 mol% of camphoric subunit does not cause a further improvement of gas barrier performance. One should consider that the camphoric subunit is particularly sterically hindered due to the presence of three substituent methyl groups in the camphoric ring, making the eventual camphoric ring stacking less effective. Furthermore, if we consider the complete exclusion of the counit from the crystal lattice (i.e., all BC segments are forced into the amorphous domains), as confirmed by the applicability of the Baur’s equation, the copolymers that are the richest in the BC counits are supposed to have a less dense amorphous phase and mesophase through which gases can pass more easily. Moreover, as previously described, as the BC content rises, the molecular weight reduces. This fact, besides the mechanical properties, could also have an effect on the gas barrier properties. Finally, another parameter to consider is the 1,4-cyclohexanedicarboxylate isomerism. As known from previous studies [14], as the cis-isomer content increases, gas permeability rises (higher GTR values). Since the two isomers adopt preferentially different conformations—boat conformation for cis-isomer—the chair conformation for trans-isomer ring stacking (chair and boat) is surely more difficult and limited with the consequent detriment of gas barrier performances. The P(BCE_95_BC_5_) copolymer is the most performant among the investigated copolymers, which is characterized by the lowest cis-isomer percentage. For all of the samples under consideration, the fastest gas diffusing through the film was found to be carbon dioxide. This behavior is typical of polymers [48]. As a matter of fact, the perm-selectivity ratio (CO_2_-TR/O_2_-TR) remains almost constant for all of the materials that were analyzed (variable in the range 2.0–2.7) and are comparable to that of the PBCE homopolymer.

## 4. Conclusions

Poly(butylene *trans*-1,4-cyclohexane dicarboxylate), a potentially bio-based cycloaliphatic polyester with smart gas barrier properties was successfully copolymerized with camphor, a cheap bio-based building block. The obtained results represent further evidence that copolymerization is an efficient strategy if we want to improve the unsatisfactory properties of a polymer for a given application without affecting the already good ones. In this specific case, we aimed to reduce the rigidity of PBCE film while trying to further improve the already good gas barrier properties and maintaining the characteristic chemical and thermal stability. The goal was fully achieved.

DSC analysis indeed showed evidence that through copolymerization, it is possible to decrease the stiffness and increase the flexibility of the reference homopolymer, making the realization of flexible thin films due to the crystallinity reduction possible.

In addition, WAXS analysis showed that the introduction of a camphor co-unit (regardless of the quantity) allowed the development of a crystalline phase in the films that was different from the one that is predominant in the PBCE homopolymer and that is characterized by a lower packing degree. Furthermore, the mesophase previously identified and responsible for the good functional properties in the PBCE homopolymer was also evidenced in the copolymers.

Mechanical results indicated that very small percentages of co-units are enough to greatly improve the elongation at break, which was modest in the PBCE homopolymer; moreover, as the percentage of butylene camphorate units increased, there was a gradual decrease in Young’s modulus, indicating a progressive reduction of the final material’s stiffness.

Even the gas barrier properties significantly improved with the introduction of small percentages of co-units. This result was explained by the combination of two factors: the development of a different crystalline phase (β phase in the copolymers vs. α latex in the homopolymer) and of the mesophase, the latter favored by lower crystallinity and greater macromolecular mobility. The extent of the changes was related to the amount of camphoric acid introduced along the PBCE macromolecular chain.

Of note, for more consistent quantities of camphor moieties, a general countertrend was observed: the functional properties returning to those of the PBCE homopolymer. The copolymers richer in BC co-units were indeed characterized by elongation at break and permeability, which decreased and increased, respectively, assuming values very similar to those of the reference homopolymer. Such result was ascribed to the lower molecular weight of these copolymers because of the lower reactivity of the camphoric acid- CCOH group due to it having one and two methyl groups in the α position.

In conclusion, a very small chemical modification, as in the case of the P(BCE_95_BC_5_) copolymer, determined a consistent improvement of the unsatisfactory properties of the starting homopolymer and preserving the excellent ones, such as the thermal stability, which is a strength of PBCE and guarantees a wide workability window.

In view of food packaging applications, antibacterial properties as well as degradability studies will be addressed in the next future.

## Figures and Tables

**Table 1:** Molecular characterization data of the PBCE homopolymer and P(BCE_x_BC_y_) copolymers.

Polymer	BCE mol% (^1^H-NMR)	Cis-Isomer mol% (^1^H-NMR)	M_n_ g/mol	D
PBCE	100	4	41,000	1.4
P(BCE_95_BCE_5_)	95	6	48,300	1.5
P(BCE_90_BC_10_)	91	7	24,500	1.7
P(BCE_85_BC_15_)	84	8	28,200	1.5

**Table 2:** Thermal (TGA and DSC) and structural (WAXS) characterization data of PBCE and P(BCE_x_BC_y_) copolymers.

Polymer	T_onset_ °C	T_max_ °C	I SCAN Powders		I SCAN Films		II SCAN				X_c_ Powders %	X_c_ Films %
			T_m_ °C	ΔH_m_ J/g	T_m_ °C	ΔH_m_ J/g	T_g_ °C	Δc_p_ J/g·°C	T_m_ °C	ΔH_m_ J/g		
PBCE	395	418	54	1.94	53	1.32	16	0.124	166	29	24	23
			167	40	166	30						
P(BCE_95_BC_5_)	392	417	50	1.98	47	1.37	12	0.141	153	22	9	20
			152	32	153	26						
P(BCE_90_BC_10_)	391	417	42	1.19	47	1.54	8	0.173	141	19	9	21
			141	25	140	21						
P(BCE_85_BC_15_)	385	416	44	1.11	47	1.88	7	0.173	132	18	5	18
			130	24	131	20						

**Table 3:** Mechanical and gas barrier data of PBCE homopolymer and P(BCE_x_BC_y_) copolymers.

Polymer	E (MPa)	σ_Y_ (MPa)	ε_Y_ (%)	σ_B_ (MPa)	ε_B_ (%)	GTR-O_2_ (cm^3^cm/m^2^ d atm)	GTR-CO_2_ (cm^3^cm/m^2^ d atm)
PBCE	560 ± 19	-	-	27 ± 2	33 ± 5	2.54	5.34
P(BCE_95_BC_5_)	492 ± 31	24 ± 1	24 ± 6	22 ± 4	250 ± 30	0.93	2.54
P(BCE_90_BC_10_)	391 ± 24	-	-	18 ± 2	20 ± 3	1.84	4.61
P(BCE_85_BC_15_)	326 ± 21	-	-	17 ± 3	22 ± 4	2.86	5.83
